# Development and Mechanical Analysis of Geopolymers Formed with Mining Residue and Fly Ash from Municipal Solid Waste Incineration Obtained After the Neutralisation Stage

**DOI:** 10.3390/polym17121704

**Published:** 2025-06-19

**Authors:** Antonia Terrones-Saeta, Juan María Terrones-Saeta, Jorge Suárez-Macías, Francisco Javier Iglesias-Godino, Francisco Antonio Corpas-Iglesias

**Affiliations:** 1 Research Group TEP-222 “Materials and Mining Engineering”, Higher Polytechnic School of Linares, Scientific and Technological Campus of Linares, University of Jaén, 23700 Linares, Spain; figodino@ujaen.es (F.J.I.-G.); facorpas@ujaen.es (F.A.C.-I.); 2 Department of Industrial and Civil Engineering, Campus Bahía de Algeciras, University of Cádiz (UCA), Avda. Ramón Puyol, 11201 Algeciras, Spain; juanmaria.terrones@uca.es; 3 Escuela de Ingeniería, Arquitectura y Diseño, Universidad Alfonso X el Sabio (UAX), Avenida de la Universidad, 1, Villanueva de la Cañada, 28691 Madrid, Spain; jsuarmac@uax.es

**Keywords:** geopolymers, potentially toxic elements, mining residue, energy source waste, circular economy mechanical analysis, sustainability, construction materials

## Abstract

Renewable energy sources are presented as a key solution to today’s energy needs, but they also generate waste that can have a negative impact on the environment. In particular, fly ash from the incineration of municipal solid waste (MSW), classified as hazardous by European regulations, is often deposited in landfills due to its lack of usefulness. This research proposes its valorisation in geopolymers, combining it with mining to create a sustainable material with a high industrial waste content. Firstly, all the wastes involved were characterised, which allowed for the development of a high-quality geopolymer from mining residue activated with 5% NaOH. This material was enriched with up to 50% fly ash (in increasing percentages) with the aim of making it inert, retaining it in the geopolymer matrix, and observing its effect on the final material. The physical and mechanical properties of the geopolymers obtained were evaluated, demonstrating that they do not produce contaminating leachates. The results indicate the feasibility of developing a geopolymer with up to 20% fly ash, obtaining a building material comparable to traditional ceramics, suitable for commercialisation, with a lower environmental impact and in line with the principles of the circular economy.

## 1. Introduction

The global energy crisis has intensified in recent decades due to the growing demand for energy and the progressive depletion of fossil fuels, such as oil, coal, and natural gas [1]. As the global population increases and economies develop, the demand for electricity has increased exponentially, leading to increased exploitation of these non-renewable resources [2]. However, this exploitation has resulted in significant environmental consequences, including climate change and pollution, which highlights the urgency of adopting more sustainable energy sources [3]. In response to this challenge, renewable energies, including solar, wind, hydro, and biomass, have emerged as viable alternatives to meet global energy demand while reducing greenhouse gas emissions [4].

Among renewable energy sources, biomass is distinguished by its capacity to provide sustainable energy and to reduce dependence on fossil fuels [5]. In Spain, biomass has become a particularly salient topic due to the abundance of agricultural, forestry, and urban waste that can be utilised as a raw material. In 2020, energy production from municipal solid waste (MSW) incineration in Spain constituted a significant proportion of the country’s renewable energy mix [6]. This process not only contributes to electricity generation, but also facilitates waste management by transforming materials that would otherwise be destined for landfill into a useful energy source [7,8].

However, the generation of solid wastes, such as fly ash and bottom ash, represents a significant environmental challenge [9]. The ashes in question contain heavy metals and other pollutants that, in the absence of appropriate management, have the potential to leach into the soil and subsequently contaminate surrounding water bodies [10,11]. Nevertheless, recent research has investigated the potential for the reuse of these wastes in the manufacture of new materials, such as additives in cements and concretes, which could mitigate their environmental impact and provide added value [12,13,14]. A number of studies have demonstrated that the use of biomass bottom ash can enhance the mechanical properties and durability of building materials [15,16,17,18], opening up new opportunities for the circular economy in the energy and construction sectors. However, fly ash generated by municipal solid waste incineration is currently disposed of in landfills, without any economically viable recovery options and at an environmental cost.

On the other hand, the construction sector is one of the world’s largest consumers of raw materials, which has a significant impact on the environment. The extraction, processing, and transportation of materials such as cement, steel, and aggregates not only require large amounts of energy, but also contribute to environmental degradation and greenhouse gas emissions. In fact, cement production, which is fundamental to construction, is responsible for around 8% of global CO_2_ emissions [19]. In addition, the sector consumes around 50% of extracted natural resources, making it one of the largest contributors to resource depletion and waste generation [20]. This situation has highlighted the need to look for sustainable alternatives that minimise the environmental impacts associated with construction [21,22,23].

In response to growing concerns about sustainability, new building materials have been developed that incorporate industrial and agricultural waste, helping to reduce the carbon footprint and conserve natural resources. For example, fly ash, blast furnace slag, glass waste, recycled tyres, and demolition waste have been used to create more sustainable building materials [24,25,26,27]. These materials not only help to reduce the amount of waste sent to landfill, but also improve the technical properties of the end products, such as durability, strength, and thermal insulation [28,29,30]. In addition, the use of waste in the production of building materials can reduce the demand for virgin raw materials, which in turn reduces the pressure on ecosystems and the energy required for production [31,32,33].

Along with the most innovative sustainable materials are geopolymers, which were discovered by the French scientist Joseph Davidovits in the 1970s [34]. Geopolymers are inorganic materials produced by alkaline activation of aluminosilicates, such as coal fly ash, metakaolin, and blast furnace slag [35,36]. This chemical process, which takes place at low temperatures, results in a material with similar or superior properties to those of traditional Portland cement, but with a significantly lower carbon footprint [37]. Success stories in the use of geopolymers include the production of concretes and mortars with improved properties, such as high compressive strength, resistance to aggressive conditions, and better thermal performance [38,39]. Furthermore, by using industrial waste as a raw material, geopolymers contribute to waste reduction and by-product valorisation, which strengthens their position as an environmentally favourable solution compared with other conventional materials [40,41].

Based on this, this research proposes the development of a geopolymeric material using a mining residue as a source of aluminosilicates. This geopolymer was added with fly ash from the incineration of municipal solid waste, considered hazardous waste and currently of no use. More specifically, the fly ash used in this research came from the gas neutralisation stage (hereafter FAN).

To this end, the highest-quality geopolymer made from the mining residue and various NaOH solutions was initially studied and then the ashes under study were added to it. The samples thus manufactured were physically and mechanically evaluated to determine whether this material could become a substitute for the ceramic material. At the same time, and in order to determine that there was no environmental impact with this new material, leachate tests were carried out.

## 2. Materials and Methods

The materials used in this research, as well as the tests that make up its scientific methodology, are presented in the following sections.

### 2.1. Materials

The materials used in this research are mainly industrial and mining residue. On the one hand, mining residue was used as the basis for the formation of the geopolymer, which was activated with sodium hydroxide (NaOH) and designed to incorporate ash from municipal solid waste (MSW) incineration. These wastes were obtained through a rigorous sampling process to ensure that the sample collected was representative of the total waste production. The waste was transferred undisturbed for characterisation and subsequent use in the analytical facilities. The following sections describe the materials in more detail, indicating their nature, generation process, origin, and key characteristics.

#### 2.1.1. Fly Ash Produced by the Incineration of Municipal Solid Waste After the Neutralisation Stage (FAN)

Fly ash, unlike bottom ash, is generated during gas treatment in municipal solid waste incineration. Fly ash is a direct by-product of the gas treatment process aimed at ‘cleaning’ the gas before it is released into the atmosphere, thus capturing all potentially toxic elements and chemical compounds present in the gases produced.

**Figure 1:**
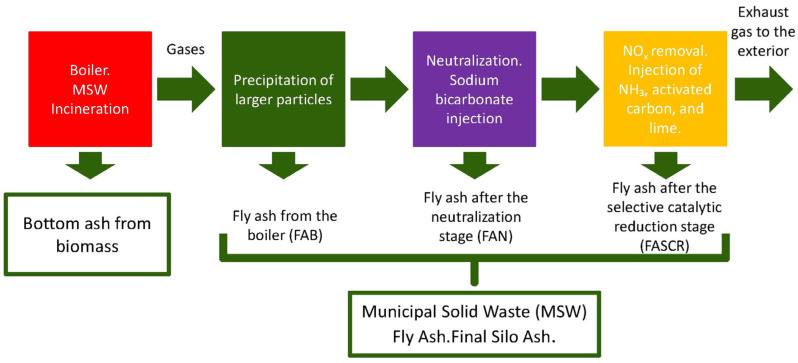
Fly ash formation process.

Gas treatment at the plant located in southern Spain, Andalusia, is carried out in several stages, as illustrated in Figure 1.

As can be seen in Figure 1, initially, biomass bottom ash is obtained, while the gases generated during municipal solid waste incineration undergo treatment. In the first stage of this treatment, the boiler fly ash, which accounts for the largest particles, is collected by gravity. Subsequently, the gases undergo a neutralisation stage, where sodium bicarbonate is injected to remove acidic compounds. The waste resulting from this stage is filtered, obtaining the neutralisation fly ash (FAN), which is used in this research. After neutralisation, the gases go through a treatment for the elimination of nitrogen oxides (NOx), which consists of the injection of ammonia, followed by the injection of lime and activated carbon. This selective catalytic reduction process leads to the formation of fly ash called FASCR.

The gas neutralisation stage, which produces the FAN ash under study, is an effective strategy for treating and reducing acid gases during municipal solid waste incineration, protecting both the environment and the equipment involved in the operation. At this stage, sodium bicarbonate injection is a technique commonly used in gas treatment plants to neutralise acids such as sulphur dioxide (SO_2_) or hydrochloric acid (HCl). This technique is preferred because bicarbonate is a safe and non-corrosive reagent for equipment and personnel, as well as being relatively inexpensive and easy to handle.

Sodium bicarbonate (NaHCO_3_) reacts with the acids present in the exhaust gases to form salts, water, and carbon dioxide (CO_2_). For example

With sulphur dioxide:

SO_2_ + 2NaHCO_3_ → Na_2_SO_3_ + 2CO_2_ + 2H_2_O (1)

With hydrochloric acid:

HCl + NaHCO_3_ → NaCl + CO_2_ + H_2_O (2)

These reactions help to reduce the acidity of the gases, transforming the acidic compounds into less harmful and more manageable products.

Finally, the ashes obtained after the neutralisation stage were collected over time and it was found that the variation in their chemical and physical properties remained constant within certain ranges. The representative sample was taken in accordance with current regulations and was not subjected to any further treatment, so the sample was used in its unaltered state in this study.

#### 2.1.2. Mining Residue from the San Ignacio Mine

**Figure 2:**
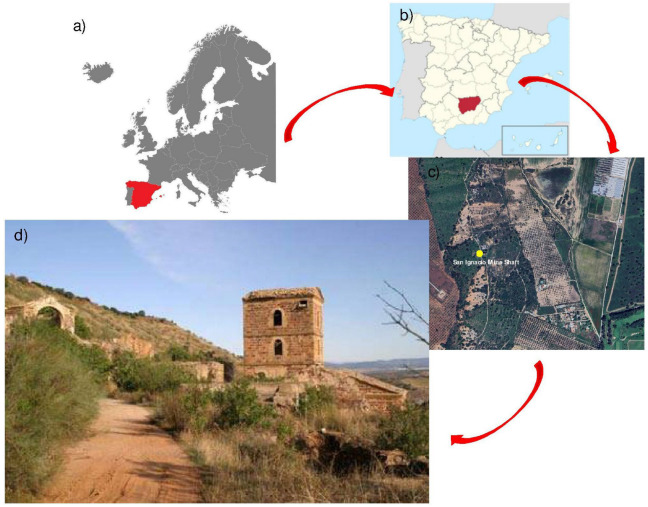
Location of the San Ignacio Mine and the mining residue produced during its production. (**a**) Location of Spanish mining, (**b**) Location of the mining district of Linares, (**c**) Location of the San Ignacio Mine, and (**d**) Image of the San Ignacio Mine waste deposits and the mine well (traditional vertical access shaft).

The mining residue used in this research originates from the dumps of the San Ignacio Mine, located in the historic mining district of Linares, Jaén, Spain. The San Ignacio Mine represents an emblematic case of the intense mining activity that characterised the region during the 19th and 20th centuries. This district, one of the most relevant in Europe for lead production, hosted numerous mining operations, with the San Ignacio Mine being one of the most prominent, as illustrated in Figure 2.

The San Ignacio Mine is located in a geological environment marked by fold structures and faults, which favoured the accumulation of minerals such as galena (PbS), the main lead ore. Mining activity in this area focused on the extraction of galena due to its high grade, which made it a resource of great economic value.

The impact of mining at the San Ignacio Mine has left a significant legacy of residue, known as dumps, from the extraction and processing of the ore. These dumps are predominantly composed of waste rock, low-grade ore fragments, and by-products of ore beneficiation.

For the present research, mining residue from the San Ignacio Mine waste dumps was used in the formulation of the geopolymer. This waste has a real density of 2.61 Tn/m^3^, comparable to that of conventional aggregates or clays. Furthermore, the content of elements with lower atomic weights, such as carbon, nitrogen, and hydrogen, is very low, also indicating a low organic matter content, as detailed in Table 1.

The elemental composition of the mine waste, detailed in Table 2, reveals a high percentage of silica, which is consistent with the geology of the region. The presence of iron in smaller proportions is responsible for the reddish colour of the waste, while aluminium is associated with the predominant granite in the area. It is relevant to highlight the low concentration of lead and other metals, given that the waste is generated after the extraction of the elements of interest.

The low concentration of metals in the waste, combined with a high ratio of silicon to aluminium and a significant amount of iron, makes it an ideal material for the production of aluminosilicates. These aluminosilicates are alkaline-activated to develop the geopolymer to incorporate the fly ash (FAN). To this end, the mining residue was obtained through a rigorous sampling process to ensure a representative sample from the San Ignacio waste dumps. The sample was then crushed to a particle size of less than 0.25 mm and oven-dried to remove residual moisture.

### 2.2. Methodology

This section details all the tests that were carried out in a structured way to develop the methodology proposed in this research.

#### 2.2.1. Physical and Chemical Characterisation of Fly Ash Produced by Municipal Solid Waste Incineration After the Neutralisation Stage (FAN)

The use of fly ash (FAN) in the production of sustainable building materials requires thorough characterisation to assess its feasibility and minimise potential environmental risks. A series of physical and chemical tests must be carried out to ensure the compatibility of FAN with other materials and to avoid potential contamination problems. The procedures used in this research are described below.

Physical testing of FAN included particle density determination and laser particle size analysis. The determination of the particle density, according to the UNE-EN 1097-7 standard [42], was carried out to assess the compatibility of the FAN with the other components of the geopolymer, since a significant variation in density could affect the mixing process, causing segregations that would affect the quality of the final material. Laser particle size analysis was carried out using the commercial Mastersizer 2000LF from Malvern Instruments (Mastersizer 2000LF, Malvern Instruments, Malvern, UK). This analysis allowed for the identification of the predominant particle size in the ashes to assess their environmental hazard and ease of handling in the formulation of geopolymers.

In order to determine the chemical composition of the FAN and its potential environmental impact, several chemical tests were carried out. The first test was elemental analysis, which measures the percentages of carbon, nitrogen, hydrogen, and sulphur present in the sample by burning at a temperature of 1000 ± 5 °C and analysing the gases produced. This test is essential for organic materials and chemical compounds containing volatile elements. The test was carried out using LECO’s TruSpec Micro (TruSpec Micro, LECO, St. Joseph, MI, USA).

The detailed elemental composition of the FAN were determined by X-ray fluorescence (XRF). This test identifies the chemical elements with the highest atomic weight present in the sample and their relative proportion, providing crucial information on possible contaminants that could affect the final product. The XRF test was carried out with Thermo Fisher’s ADVANT’XP+ equipment (ADVANT’XP+, Thermo Fisher, Waltham, MA, USA).

Finally, to evaluate the reactivity of the elements present, an X-ray diffraction (XRD) analysis was performed to identify the main chemical compounds or phases in the FAN. This analysis was performed with PANalytical’s X’Pert PRO model equipment (X’Pert PRO, PANalytical, Malvern, UK), allowing for a deeper understanding of the compounds present and their behaviour in the formation of geopolymers.

#### 2.2.2. Conforming of Geopolymer Samples with the Mining Residue and FAN—Physical and Mechanical Tests

As mentioned above, the aim of this research is to integrate fly ash (FAN) from municipal solid waste incineration into a sustainable geopolymeric material. This approach aims not only to retain the pollutants present in the FAN, but also to use these ashes as a raw material for the production of new building materials.

The first step was to evaluate the geopolymer that would act as the host matrix for the FAN. This geopolymer was formed from the previously described mining residue, which serves as a source of aluminosilicates, and was alkaline-activated using a sodium hydroxide (NaOH) solution. The choice of NaOH concentration in the solution is critical to the quality of the final geopolymer. Therefore, tests were carried out with different concentrations of NaOH dissolved in demineralised water to determine the optimum concentration that would afford the best properties to the geopolymer.

For this purpose, a given mass of mining residue was prepared, crushed to a suitable particle size, and 20% by mass of NaOH solution was added. The NaOH concentrations tested were 0%, 2.5%, 5%, 7%, 7.5% and 10% by mass of mining residue. The preparation consisted of dissolving the different percentages of NaOH in demineralised water and mixing it with the mining residue until complete homogenisation was achieved. This mixture was poured into a metal matrix measuring 60 mm by 30 mm and compacted at a pressure of 20 MPa. The samples obtained from the different concentrations of NaOH were extracted from the metal matrix and left to cure for 24 h at 20 ± 1 °C. They were then dried for 24 h at 60 ± 1 °C to remove any residual moisture.

In order to determine the quality of the geopolymer according to the different NaOH concentrations, a compressive strength test was carried out according to the UNE-EN 772-1 standard [43]. This test made it possible to determine which concentration of NaOH afforded the highest compressive strength and, therefore, the best quality geopolymer for incorporating FAN.

On the basis of the results obtained, the optimum percentage of NaOH that afforded the highest compressive strength was determined. Subsequently, different percentages of FAN were incorporated into the geopolymer to evaluate their effect on the geopolymer matrix through various physical and mechanical tests. The aim was to determine the maximum percentage of FAN that could be added without degrading the final geopolymer material.

The samples produced with the different proportions of geopolymer and FAN are detailed in Table 3, which provides a summary of the compositions and conditions of the blends evaluated in this research.

The fabrication of the test samples for the geopolymer and fly ash (FAN) series, described above, follows a similar process to the one used for the determination of the optimal geopolymer mechanically with the mining residue. Initially, the appropriate amounts of geopolymer were mixed with the previously evaluated optimal NaOH solution. Subsequently, the corresponding mass percentage of FAN was added. The mixture was stirred until complete homogenisation of the material was achieved.

Once the homogeneous mixture was obtained, it was deposited in a metal matrix with dimensions of 60 mm by 30 mm and compacted at a pressure of 20 MPa. After compaction, the sample was removed from the matrix and left to cure at 20 ± 1 °C for 24 h. Subsequently, the sample was dried for 24 h at 60 ± 1 °C before proceeding with the physical and mechanical tests necessary to evaluate the impact of the FANs on the geopolymer material. This process was repeated for each of the geopolymer and FAN series, resulting in a total of six samples for each family.

The FAN-formed samples from each geopolymer family were subjected to a series of physical tests. The first test wad the capillary water absorption test, carried out in accordance with the UNE-EN 772-11 standard [44]. This test made it possible to determine the percentage of water that the geopolymer material could absorb by capillarity during one minute of partial immersion. This analysis is crucial for materials exposed to the elements or to humid soils. In addition, the cold water absorption test was carried out according to the UNE-EN 772-21 standard [45], which measured the percentage of water absorbed by the geopolymer material during a 24 h immersion. Finally, the open porosity and bulk density tests were carried out in accordance with the UNE-EN 772-4 standard [46]. These tests allowed for the quality of the geopolymer formed with the FANs to be evaluated by determining the number of pores and the density of the material, both critical factors for the strength of the geopolymer.

It is important to note that the colour of the construction materials can be a limiting factor. The incorporation of wastes can significantly affect the final colour of the material, which, although not directly influencing its mechanical behaviour, is relevant to its marketability. In this research, any subjective judgement on colour was avoided and an objective measurement of the chromatic property was carried out using a colourimeter, model PCE-RGB-2 (PCE, Meschede, Germany).

Finally, given that the geopolymeric materials developed are intended to replace ceramic materials and concrete parts in construction, it is essential to evaluate the variation in compressive strength due to the incorporation of FAN into the matrix. For this purpose, the compressive strength test according to UNE-EN 772-1 was carried out on all the samples. This analysis made it possible to determine the maximum percentage of FAN that can be added to the geopolymer developed without compromising its structural function.

#### 2.2.3. Leachate Testing of FAN Formed Geopolymeric Samples

The utilisation of mining and industrial waste in the creation of new sustainable materials presents a multitude of environmental and economic benefits. These advantages include a reduction in the extraction of virgin raw materials, waste deposition in landfills, and the capacity of the developed materials to retain polluting chemical elements, thus minimising potential environmental contamination. In this context, one of our primary objectives is to prevent the generation of polluting leachates from the developed geopolymeric material. It is therefore essential to evaluate the leachates from the various geopolymer series containing fly ash (FAN).

To conduct this assessment, we employed the Leachability Capacity for Hazard Characterisation (TCLP) test, an internationally recognised standard technique for the assessment of leachates in construction materials. The TCLP test is a widely used method among researchers for analysing the leaching potential of contaminants, and it is applicable in a variety of regulatory contexts.

The TCLP procedure commenced with the grinding of the geopolymer samples to a particle size of less than 10 mm. The samples were then combined with a leaching solution at a 1:20 solid:liquid ratio. The mixture was stirred for 18 ± 2 h at a controlled temperature of 22 ± 3 °C. The leaching solution was prepared by adding 5.7 mL of glacial acetic acid and 64.3 mL of a sodium hydroxide solution (1 N) to 1 L of distilled water. Following the stirring process, the mixture was filtered using a glass fibre filter with an effective pore size of 0.7 μm. The filtrate was acidified with nitric acid to pH 2 and analysed by inductively coupled plasma–mass spectrometry using model 7900 (Agilent Technologies, Santa Clara, CA, USA).

Table 4 lists the chemical elements considered potentially toxic by the United States Environmental Protection Agency (US EPA) and the maximum allowable concentrations in the leachate, according to the TCLP method.

In this way, the analysis of the leachates made it possible to assess the leaching of potentially toxic elements in the different geopolymer series. This assessment is crucial to determine the maximum percentage of FAN that can be added to the geopolymer without compromising environmental safety.

## 3. Results and Discussion

The results obtained from the tests described in the methodology are presented in this section in a structured manner. Partial conclusions derived from the experimental data are included and the decisions made during the research process to achieve the stated objectives are detailed. This approach allows for a clear and systematic evaluation of the findings and facilitates the interpretation of their impact on the research.

### 3.1. Physical and Chemical Characterisation of Fly Ash Produced by Municipal Solid Waste Incineration After the Neutralisation Stage (FAN)

The physical and chemical properties of MSW incineration ashes were first determined to evaluate their characteristics and the possible problems with incorporating them into geopolymers. The particle density of the FAN was found to be 2.70 Tn/m^3^. This is similar to the particle density of normal aggregates and materials, which is usually around 2.65 Tn/m^3^. The similar density means there should be no problems mixing the FAN and mine waste.

**Figure 3:**
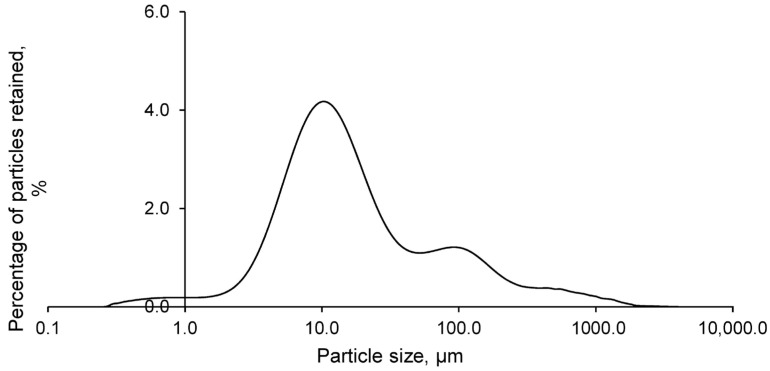
Particle size distribution of ash from municipal solid waste incineration.

The particle size analysis of the FANs, as shown in Figure 3, revealed that the particle size varied between 5 and 500 μm. However, most of the particles were concentrated around 10 μm. This small particle size contributed to the hazardousness of HABs, as it facilitated their dispersion and transport into the environment. However, this characteristic also favoured the retention of the residue in the geopolymer matrix, benefiting the material forming process.

Additionally, an elemental analysis was performed to determine the percentages of carbon, nitrogen, and hydrogen present in the FAN. The results of this test are presented in Table 5. This analysis is fundamental for the chemical characterisation of the FANs and provides key information on their chemical composition and potential environmental impact.

Analysis of fly ash (FAN) revealed very low contents of carbon, nitrogen, and hydrogen, indicating a low percentage of organic matter and hydrated compounds in the ash. The virtual absence of carbon and hydrogen suggests that the FAN does not contain carbonate or hydrated compounds, although other volatile components may be present.

To determine the composition of the elements present in the higher atomic weight FAN, an X-ray fluorescence (XRF) assay was performed. The results of the analysis are presented in Table 6.

The X-ray fluorescence assay showed a significantly high ignition loss of 77.34%, indicating an unstable nature of the ash, with the presence of volatile elements and unstable chemical compounds that were transformed during the analysis. This high instability suggests a potential environmental risk due to the ability of FAN to disperse in aquatic environments.

Among the chemical elements detected, chlorine is present in the highest proportion. This element may negatively affect the quality of the geopolymer and requires further evaluation. Sodium, potassium, sulphur, calcium and silicon were also detected, which is expected given the role of the neutralisation system and the injection of sodium bicarbonate in the incineration process. On the other hand, metals such as zinc, tin and lead are present in smaller proportions, derived from the incinerated materials. The presence of these potentially contaminating metals in FAN underscores the need to carefully evaluate the geopolymeric material incorporated in FAN to ensure that hazardous leachates are not produced.

**Figure 4:**
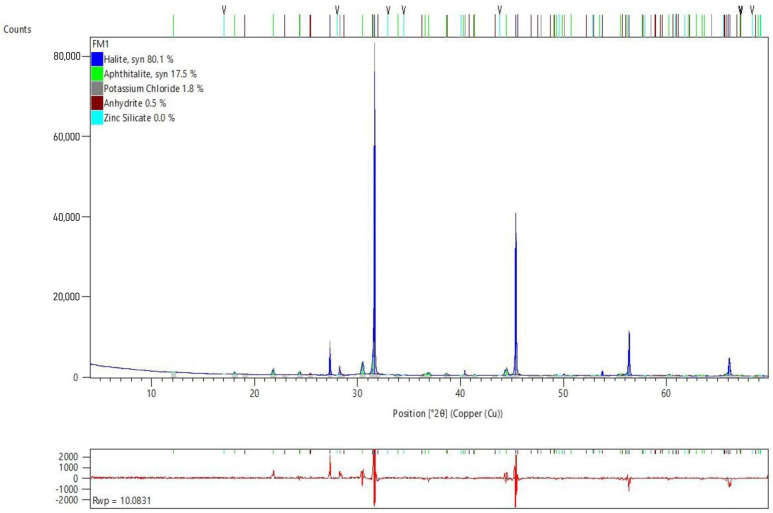
X-ray diffraction test of fly ash produced by the combustion of municipal solid waste after the neutralisation stage.

In order to determine the chemical compounds in which the above chemical elements are combined, as well as to evaluate their greater or lesser chemical instability, the X-ray diffraction test was performed. The results of this test are shown in Figure 4.

X-ray diffraction (XRD) analysis revealed that the predominant chemical composition of the fly ash (FAN) consists mainly of halite (NaCl) and aphthalite (K, Na)_3_Na(SO_4_)_2_. This composition is consistent with the origin of the ashes, coming from the neutralisation of gases generated during municipal solid waste incineration, in which large amounts of sulphur and chlorine are produced. Both sodium chloride (NaCl) and potassium sulphate (K_2_SO_4_) are soluble in water, and in high concentrations could modify soil and water chemistry, negatively affecting local ecosystems.

In addition to these major compounds, XRD analysis identified other compounds in smaller proportions, such as potassium chloride (KCl), anhydrite (CaSO_4_), and zinc silicate (Zn_2_(SiO_4_)). These compounds also present potential environmental problems similar to those of the main compounds detected. The presence of zinc silicate, while not extremely toxic to humans, can easily transfer to soil and surface or groundwater, causing significant environmental impacts and affecting organisms and microorganisms in these environments.

Consequently, the small particle size of FAN, together with its unstable chemical composition and the presence of potentially toxic metallic elements, classifies this ash as a hazardous waste. The main motivation of this research is to avoid the deposition of these ashes in landfills, where they could be transferred to the environment, and to ensure their safe retention in the geopolymer matrix, as detailed in later sections.

### 3.2. Conforming of Geopolymer Samples with the Mining Residue and FAN—Physical and Mechanical Tests

As detailed in the methodology, the first step in the development of the geopolymer consisted of using the mining residue as a source of aluminosilicate, which was alkaline-activated by means of sodium hydroxide solutions at different concentrations (0%, 2.5%, 5%, 7.5%, and 10%) in relation to the mass of the mining residue. To these solutions, 20% of water was added in relation to the mass of the mining residue.

**Figure 5:**
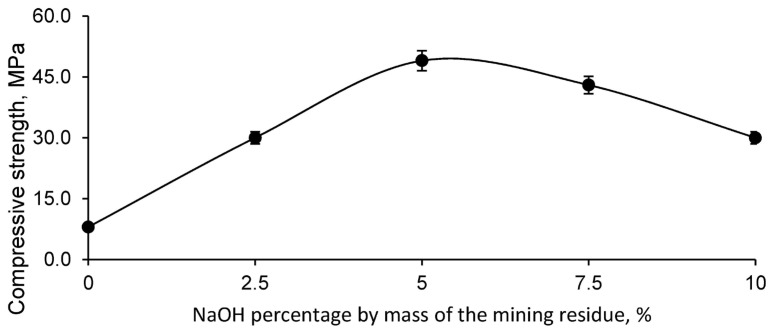
Compressive strength of the series of geopolymeric samples formed with the mining residue and with different solutions of sodium hydroxide as the alkaline activator.

The main criterion for determining the optimum geopolymer was the compressive strength, since the aim was to obtain a geopolymeric material with the highest possible strength for construction applications. The results obtained from the compressive strength test for the different geopolymer mixtures, formed with the mining residue and different concentrations of NaOH, are shown in Figure 5.

As illustrated in Figure 5, the geopolymer made from the mining residue showed the highest compressive strength when using a 5% NaOH dilution in the mass of the mining residue, with an addition of 20% water over the mass of the residue. This combination proved to be the most effective in terms of mechanical strength. Consequently, the geopolymer formulated for the incorporation of different percentages of fly ash (FAN) was based on this optimum geopolymer formulation, as detailed in Table 3.

**Figure 6:**
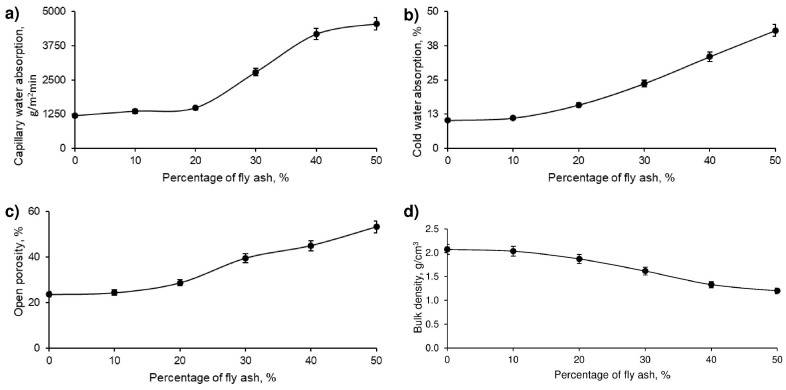
Physical properties of the samples formed with the geopolymer produced with the mining residue and different percentages of FAN, from 0% to 50%. (**a**) Capillary water absorption vs. percentage of FAN, (**b**) Cold water absorption vs. percentage of FAN, (**c**) Open porosity vs. percentage of FAN, and (**d**) Bulk density vs. percentage of FAN.

The different geopolymer formulations with various percentages of FAN, presented in Table 3, were prepared following the procedure described in the methodology. Subsequently, physical tests were carried out on the samples obtained, the results of which are shown in Figure 6. These tests made it possible to objectively evaluate the impact of the addition of FAN on the physical properties of the geopolymer.

Figure 6 illustrates that capillary water absorption increased with the increase in the percentage of fly ash (FAN) in the developed geopolymer. This increase was due to the formation of a structure with a greater number of interconnected channels in the material, which facilitates greater water absorption. This behaviour can be explained by Jurin’s law. Considering that ceramic materials in direct contact with wet soils should limit their capillary water absorption to 4000 g/m^2^-min, samples with 50% FAN exceed this threshold and would be considered unsuitable for applications in such conditions.

On the other hand, cold water absorption also increases with increasing percentage of FAN in the geopolymer. Although this property is not regulated by the standards used, it is crucial for building materials exposed to the elements, such as roof tiles, since higher water absorption can increase the weight of the material and, therefore, the structural load. It is relevant to note that the variation in cold water absorption is minimal when introducing 10% or even 20% FAN in the geopolymer.

Moreover, the porosity of the geopolymer material, as expected, increases with the percentage of FAN. This increase in porosity indicates a more open and less dense structure of the material. In construction applications for thermal and acoustic insulation, higher porosity can be advantageous, as it reduces the thermal and acoustic conductivity of the material.

Finally, in correlation with the previous results, it is observed that the bulk density decreases as the percentage of FAN in the geopolymer increases. Since the densities of the mining residue and FAN are similar, this decrease in density reflects a more porous structure of the material, which may negatively affect its strength. However, it is important to note that the variation in bulk density is reduced in geopolymer series with 10% and even 20% FAN.

**Figure 7:**
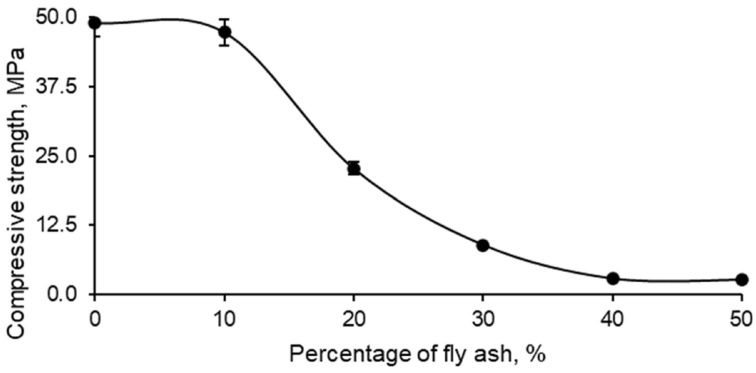
Compressive strength of the different series of geopolymers made with the mining residue and increasing percentages of FAN.

In relation to the evaluation of the strength of the different series of samples formed with geopolymer and FAN, the results obtained from the compressive strength test are shown in Figure 7.

The compressive strength of geopolymers produced with fly ash (FAN) showed a clear tendency to decrease with increasing percentage of FAN. This behaviour can be attributed to the characteristics observed in previous tests, where it was established that greater water absorption was due to an increase in porosity, which in turn decreased the density of the material and, consequently, its compressive strength. Figure 7 presents this decrease in compressive strength as a function of the percentage of FAN incorporated.

In particular, it is observed that the addition of 10% FAN to the geopolymer did not cause a significant variation in compressive strength. This indicates that the geopolymer material retained acceptable properties in terms of strength, water absorption, density, and porosity. In contrast, the addition of 20% FAN resulted in a drastic 50% reduction in compressive strength, which implied a notable loss of this fundamental mechanical property. The addition of higher percentages of FAN, such as 30%, 40% and 50%, led to a material with practically zero strength, making it unsuitable for structural applications. However, these percentages could be useful for alternative applications such as thermal and acoustic insulation, although these uses are not part of the scope of this research.

Finally, in order to accurately determine the colour of the different series of samples, chromatic coordinate measurements were carried out using the equipment specified in the methodology. The results of these measurements are presented in Table 7.

**Figure 8:**
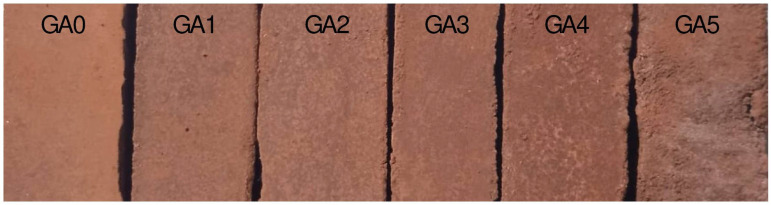
Image of the samples and the colour variation in the geopolymer due to the addition of FAN in its matrix. From left to right, GA0, GA1, GA2, GA3, GA4, and GA5 family.

As in previous properties, it can be seen how the coordinates of the primary colours varied with the additions of FAN, creating a chromatic of colours that, far from being subjectively valued, are simply presented in Figure 8.

### 3.3. Leachate Testing of FAN Formed Geopolymeric Samples

The objective of this research is the valorisation of fly ash (FAN) in the development of new sustainable materials for construction. Up to this point, the feasibility and impact of incorporating FAN in geopolymeric materials have been evaluated. However, it is essential to ensure that these ashes are adequately retained in the material matrix and to avoid leaching of contaminants that may cause adverse environmental impacts. Therefore, it is crucial to analyse leachates from geopolymeric materials made with FAN and ensure that no contaminant leachates are generated.

**Figure 9:**
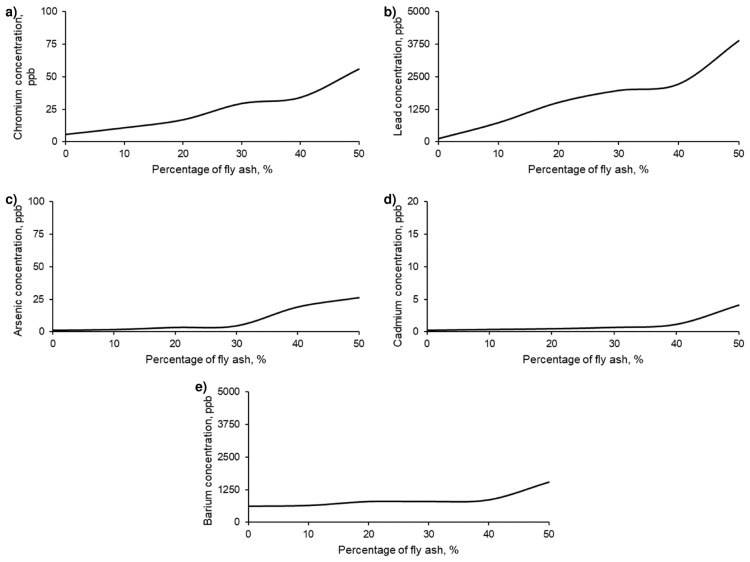
Concentrations of chromium (**a**), lead (**b**), arsenic (**c**), cadmium (**d**), and barium (**e**) in the leachates obtained according to the TCLP test of the different series of geopolymers formed with different percentages of FAN.

For all series of samples, the leachates were analysed according to the regulations and limitations specified in the methodology. The results of these analyses are presented in Figure 9.

## 4. Conclusions

The results and discussion of the tests performed permit an evaluation of the fulfilment of the research objective. The principal aim was to develop a geopolymeric material utilising mining residue, which could incorporate fly ash from the incineration of municipal solid waste after its neutralisation, with physical and mechanical properties comparable to those of traditional ceramic materials. From the tests conducted, partial conclusions were derived that are fundamental for the interpretation and determination of the final conclusion of this research.

- The mining residue from the lead mine dump in the Linares mining district has a chemical composition suitable for being a source of aluminosilicates in geopolymeric materials;
- Incineration fly ash (FAN) has a small particle size, high solubility, and significant metal content, which classifies it as a hazardous waste with environmental risk if not properly treated;
- It is feasible to formulate geopolymers with the mining residue alkaline-activated with a 5% NaOH solution. These geopolymers show a compressive strength of 50 MPa and physical properties suitable as a substitute for construction ceramics (bricks, tiles);
- The incorporation of FAN in the geopolymeric material decreases the physical and mechanical quality, increasing the porosity and reducing the density and compressive strength. However, additions of up to 20% FAN have a lesser effect than higher concentrations;
- Geopolymeric samples with 40% and 50% FAN have unacceptable compressive strengths according to standards, making them unviable for commercialisation. However, additions of 10% and 20% show acceptable values, exceeding the resistance limits;
- Geopolymer leachates with different percentages of FAN show concentrations of potentially toxic elements, according to US-EPA regulations, below the maximum permitted levels.

Based on the partial conclusions obtained and the tests performed, it can be affirmed that it is feasible to manufacture geopolymers using mining residues incorporating FAN in their matrix, achieving sustainable construction materials with acceptable physical, chemical, and mechanical properties. This approach represents a clear example of circular economy, offering a viable solution for waste that would otherwise be destined for landfill, generating significant environmental and economic costs.

Looking ahead, these materials show potential for non-structural applications in the construction sector, such as blocks, tiles, pavements, or partition elements. Furthermore, this research opens the door to future lines of investigation focused on further optimising the formulation through the incorporation of additional waste streams or alternative precursors, as well as long-term durability studies, environmental exposure performance, and life-cycle assessment (LCA) to evaluate their true sustainability impact.

## Figures and Tables

**Table 1:** Elemental analysis (carbon, nitrogen, and hydrogen) of mining residue from the mining operation “San Ignacio”.

Name	Nitrogen, %	Carbon, %	Hydrogen, %
Mining residue “San Ignacio”	0.135	0.407	0.151

**Table 2:** X-ray fluorescence test of mining residue from the “San Ignacio” mining operation.

Element	Wt, %	Est. Error
Si	40.27	0.08
Fe	5.18	0.09
Al	1.63	0.05
K	1.17	0.05
Pb	0.26	0.013
Mg	0.0951	0.0068
Ti	0.0848	0.0042
Ca	0.083	0.0042
Na	0.0799	0.014
Cr	0.0494	0.0025
Px	0.0163	0.0018
Sx	0.0131	0.0009
Mn	0.0178	0.0011
Ni	0.0154	0.0015
Zr	0.0116	0.0034
W	0.0117	0.0044
Cl	0.012	0.001
Ru	0.0079	0.0023
Pt	0.0088	0.0031
Known Concentration =		0.91 LOI

**Table 3:** Composition of the different series of samples formed with the geopolymer made with the mining residue and different percentages of FAN.

Sample Series	wt.% of Geopolymer Matrix (Mining Residue + 5% NaOH Solution)	% FAN
GA0	100	0
GA1	90	10
GA2	80	20
GA3	70	30
GA4	60	40
GA5	50	50

**Table 4:** Maximum concentrations of metals or toxic elements in the leachate, according to US.EPA, obtained with the TCLP method [47].

Metals	Maximum Allowable Concentration in Leachate, ppb
Cr	5000
Pb	5000
As	5000
Cd	1000
Ba	100,000

**Table 5:** Elemental analysis (carbon, nitrogen, and hydrogen) of fly ash produced by the combustion of municipal solid waste after the neutralisation stage.

Name	Nitrogen, %	Carbon, %	Hydrogen, %
FM1	0.163	0.702	0.176

**Table 6:** X-ray fluorescence testing of fly ash produced by the combustion of municipal solid waste for electric power generation.

Element	Wt, %	Est. Error
Cl	8.96	0.14
Na	5.33	0.1
K	1.89	0.06
Sx	0.651	0.03
Zn	0.646	0.032
Ca	0.312	0.016
Si	0.155	0.0077
Pb	0.232	0.012
Sn	0.112	0.0056
Br	0.124	0.0062
Sb	0.0793	0.004
Fe	0.0604	0.003
Mg	0.0492	0.0025
Cu	0.0436	0.0022
Ba	0.0384	0.0044
Cd	0.0348	0.0017
Px	0.0136	0.0007
Ti	0.0127	0.0006
Al	0.0107	0.0012
I	0.0176	0.003
Cs	0.0099	0.0032
Known Concentration		77.34 LOI

**Table 7:** Colour value of the different series of samples according to the primary colours (red, green, blue, green, blue, green, and blue).

Samples Series	% Fly Ash in Geopolymer	Red	Green	Blue
GA0	0	136	76	60
GA1	10	130	70	56
GA2	20	144	70	56
GA3	30	123	66	50
GA4	40	119	65	51
GA5	50	163	92	73

## Data Availability

Data is contained within the article.
